# Estimation of a Common Effect Parameter from Follow-Up Data When There Is No Mechanistic Interaction

**DOI:** 10.1371/journal.pone.0086374

**Published:** 2014-01-20

**Authors:** Wen-Chung Lee

**Affiliations:** Research Center for Genes, Environment and Human Health, and Institute of Epidemiology and Preventive Medicine, College of Public Health, National Taiwan University, Taipei, Taiwan; University of Illinois-Chicago, United States of America

## Abstract

In a stratified analysis, the results from different strata if homogeneity assumption is met are pooled together to obtain a single summary estimate for the common effect parameter. However, the effect can appear homogeneous across strata using one measure but heterogeneous using another. Consequently, two researchers analyzing the same data can arrive at conflicting conclusions if they use different effect measures. In this paper, the author draws on the sufficient component cause model to develop a stratified-analysis method regarding a particular effect measure, the ‘peril ratio’. When there is no mechanistic interaction between the exposure under study and the stratifying variable (i.e., when they do not work together to complete any sufficient cause), the peril ratio is constant across strata. The author presents formulas for the estimation of such a common peril ratio. Three real data are re-analyzed for illustration. When the data is consistent with peril-ratio homogeneity in a stratified analysis, researchers can use the formulas in this paper to pool the strata.

## Introduction

A central issue in epidemiology is characterizing the relationship between exposure and disease. As many other factors may confound or modify the effect of exposure under study, epidemiologists often need to perform a stratified analysis of these confounders/modifiers. On the one hand, if heterogeneity of exposure effects are present (i.e., the effects are different across strata), then we report the stratum-specific estimates separately. On the other hand, if the data is consistent with homogeneity, then we pool the results from different strata to obtain a single summary estimate for the common effect parameter of the exposure [1].

However, the effect of an exposure can either be measured in a ratio scale, e.g., risk ratio, odds ratio and rate ratio, or in a difference scale, e.g., risk difference, odds difference and rate difference [1]. No one scale is better than the others and so universally endorsed. Worse, an effect can appear homogeneous across strata when using one measure and heterogeneous when using another. Consequently, if using different measure, two researchers analyzing the same data can arrive at conflicting conclusions, which is certainly undesirable.

The sufficient component cause model [1]–[13] can help to resolve this conflict. A sufficient cause contains a combination of component causes. There may be many classes of sufficient causes for a disease, and any class with all of its components completed is sufficient to cause the disease. When there is no mechanistic interaction between the exposure under study and the stratifying variable (i.e., when they do not work together to complete any sufficient cause), a particular effect measure, the ‘peril ratio’, will be constant across strata [13]. In this paper, I present formulas for the estimation of such a common peril ratio. Three real data will be re-analyzed for illustration.

## Methods

Consider a dichotomous exposure and disease in the follow-up of a population in a certain time interval. The exposure status is assumed to be time-invariant, and the follow-up, to be without loss to follow up and competing death. A stratified analysis is to be performed based on a stratification variable with a total of 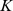
(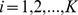) strata. Table 1 presents the data layout for the 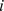 ^th^ stratum.

**Table 1:** Data layout for the *i*th stratum.

	Non-diseased	Diseased	Total
Unexposed	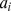	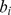	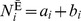
Exposed	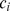	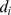	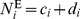
Total	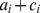	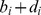	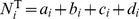

The peril ratio (PR) for the 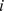 ^th^ stratum is defined as
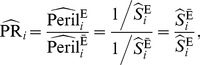
(1)where 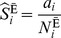 is the disease-free proportion at the 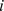 ^th^ stratum for the unexposed, and 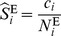 is for the exposed [13]. Note that a peril ratio is simply a risk ratio with reverse coding for both the exposure and the outcome. Therefore, formulas for risk ratio [1] can be directly applied to the peril ratio, if reverse coding is properly acknowledged. A large-sample formula for the variance of a log peril ratio is



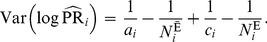
(2)Peril ratios are to be interpreted as ‘fold decreases’ [13]. Assuming that no residual confounding exists, 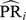 is the fold decrease in a disease-free probability for a subject at the 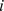 ^th^ stratum if he/she changes status from being unexposed to being exposed. Under the assumption that there is no mechanistic interaction between the exposure under study and the stratifying variable, the peril ratios are constant across strata [13]. This common peril ratio is the fold decrease in disease-free probability for anyone, regardless of the stratum, whose status changes from being unexposed to being exposed.

To estimate the common peril ratio, one can use the inverse-variance weighted Woolf-type estimator [14]:
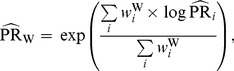
(3)where 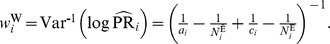 The variance of the logarithm of this pooled estimate (under the large-stratum limiting model) is [14]



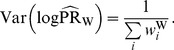
(4)Alternatively, one can use the Mantel-Haenszel estimator [15]:
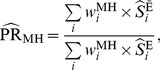
(5)where 
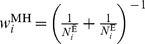
, the Mantel-Haenszel weight, is the harmonic sum of the unexposed (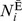) and the exposed (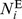) population for the 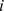 ^th^ stratum. A variance formula for the Mantel-Haenszel estimator, which is valid under both the large-stratum and sparse-data limiting models is [15]

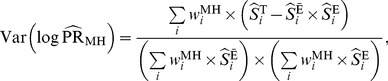
(6)where 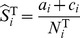 is the disease-free proportion of the 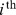 stratum.

In the above, we have invoked the assumption of peril-ratio homogeneity (equivalently, the assumption of no mechanistic interaction). In practice, this assumption needs to be checked using the data on hand. Here, I extend the PRISM (peril ratio index of synergy based on multiplicativity) test used in a previous paper [13] in order to deal with the present situation of 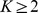. First, we calculate 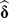, a 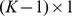 column vector of the estimates of the logPRISMs with its 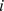 ^th^ element (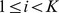) being
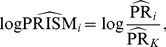
(7)and 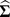, a 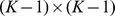 variance-covariance matrix of 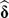 with its 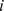 ^th^ diagonal element (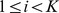) being
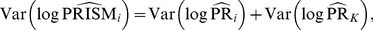
(8)and its 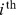 row and 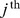 column (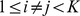) off-diagonal element,
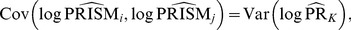
(9)respectively. Next, we calculate the following heterogeneity statistic (Het):



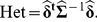
(10)Asymptotically (large-stratum limiting model), Het is distributed as a chi-square distribution with 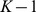 degree of freedom (df) under the null hypothesis of peril-ratio homogeneity (no mechanistic interaction).

If a summary effect measure is the desired end but the assumption of peril-ratio homogeneity fails, then one can resort to standardization techniques [1] in order to pool the strata. In general, the resulting standardized effect measures have larger variances. Using the total population as the standard, the standardized peril ratio is calculated as
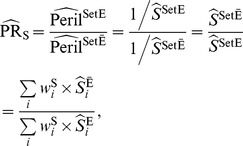
(11)where the ‘Set X’ operator dictates that the exposure status of each and every subject in the population is set to X, and the weight, 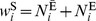, is the population size (the arithmetic sum of the unexposed and the exposed, cf., the harmonic sum in Mantel-Haenszel weight) for the 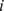 ^th^ stratum. The variance of 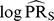 under the large-stratum limiting model is
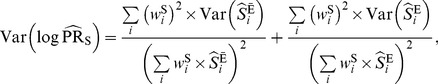
(12)with 
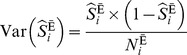
 and 
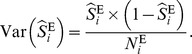
 This standardized peril ratio is what the fold decrease in the disease-free proportion for the entire population would be, if we, contra the facts, change the exposure prevalence of the population from 0% to 100%.

It is worth noting that all three summary peril ratios considered in this paper, 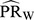, 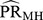 and 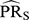, enjoy the ‘collapsibility’ property [1]; that is, the summary effect measure will fall somewhere in the middle of the stratum-specific measures. This is so because they are weighted averages of the stratum-specific peril ratios [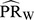: geometric average with the weights proportional to 
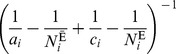
; 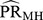: arithmetic average with the weights proportional to 
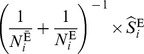
; 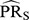: arithmetic average with the weights proportional to 
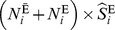
].

## Examples

In this section, three real data were re-analyzed in order to demonstrate the methodologies.

### 1. Mortality Data from All causes for Tolbutamide and Placebo Treatment Groups

The first example consists of randomized, controlled trial data comparing all-cause mortality between tolbutamide treatment and placebo groups, taken from Table 15–1 in the textbook *Modern Epidemiology* [1]. The stratifying variable is age (two strata: age<55 and age 55+). Table 2 presents the peril ratios and their 95% confidence intervals (CIs) for the two strata. The heterogeneity statistic is calculated as 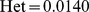. This is to be referred to a chi-square distribution with 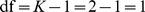, and the p-value is 0.9057.

**Table 2:** Re-analysis of the mortality data from all causes for tolbutamide and placebo treatment groups^a^.

	Surviving	Dead	Total	Peril Ratio (95% CI^b^)
Age<55				
Placebo	115	5	120	1.0000
Tobultamide	98	8	106	1.0366 (0.9704∼1.1072)
Age 55+				
Placebo	69	16	85	1.0000
Tobultamide	76	22	98	1.0467 (0.9030∼1.2134)
Total(Crude)				
Placebo	184	21	205	1.0000
Tobultamide	174	30	204	1.0523 (0.9779∼1.1324)

a from Table 15-1 in Rothman KJ, Greenland S, Lash TL (2008) Modern Epidemiology, 3rd ed. Philadelphia: Lippincott.

b CI:confidence interval.

Because the data is consistent with peril-ratio homogeneity (no mechanistic interaction between treatment and age), I pooled the two strata in order to obtain a common peril ratio: 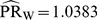 (95% CI: 0.9776∼1.1027) using Woolf’s method, or 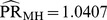 (95% CI: 0.9688∼1.1179) using the Mantel-Haenszel method. This implies a ∼4% reduction in survival for anyone, young or old, who chooses to take tolbutamide (though it is not significant, judging from the 95% CIs that cover the no-effect peril ratio of one). For this example, age is not an important confounder; the common peril ratio and the crude peril ratio (1.0523) differ very little. This is no surprise as the data is drawn from a randomized controlled trial.

### 2. Coronary Heart Disease Occurrence Data for Personality Type A and B Persons

The second example consists of cohort data comparing the occurrence of coronary heart disease (CHD) between personality type A and B persons, taken from Table 7–24 in the textbook *Statistical Analysis of Epidemiologic Data* [16]. The stratifying variable is age (a total of five strata). Table 3 presents the peril ratios and their 95% CIs for the five strata.

**Table 3:** Re-analysis of the coronary heart disease (CHD) occurrence data for personality type A and B persons^a^.

	No CHD	CHD	Total	Peril Ratio (95% CI^b^)
Age<40				
Type B	271	11	282	1.0000
Type A	241	20	261	1.0407 (0.9978 ∼ 1.0855)
Age 40–44				
Type B	574	21	595	1.0000
Type A	462	34	496	1.0357 (1.0067 ∼ 1.0655)
Age 45–49				
Type B	343	21	364	1.0000
Type A	337	49	386	1.0793 (1.0311 ∼ 1.1298)
Age 50–54				
Type B	184	17	201	1.0000
Type A	209	38	247	1.0819 (1.0110 ∼ 1.1577)
Age 55+				
Type B	114	9	123	1.0000
Type A	162	37	199	1.1385 (1.0479 ∼ 1.2369)
Total(Crude)				
Type B	1486	79	1565	1.0000
Type A	1411	178	1589	1.0693 (1.0472 ∼ 1.0919)

a from Table 7–24 in Selvin S (1991) Statistical Analysis of Epidemiologic Data. New York: Oxford University Press.

b CI: confidence interval.

Again, the data is consistent with peril-ratio homogeneity (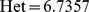 and p-value
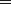
0.1505, based on a chi-square distribution with 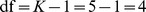). I then pooled the five strata in order to obtain a common peril ratio: 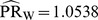 (95% CI: 1.0335∼1.0744) using Woolf’s method, or 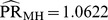 (95% CI: 1.0407∼1.0842) using the Mantel-Haenszel method. This implies a 5∼6% reduction in CHD-free probability for a type A person when compared with a type B person of the same age. This reduction is significant, judging from the 95% CIs that do not cover the no-effect peril ratio of one.

### 3. Subsequent Cocaine Use Data for Early Marijuana Users and Non-Users

The final example consists of twin follow-up data for subsequent cocaine use comparing exposed twin members (early marijuana users) with their unexposed co-twins (non marijuana users), and is taken from the paper of Cummings and McKnight [17]. Treating each twin pair (a total of 311 pairs) as one separate stratum, the data can be presented in a total of 311 ‘tables’ (see Table 4).

**Table 4:** Re-analysis of the subsequent cocaine use data for exposed twin members (early marijuana users) and their unexposed co-twins (non marijuana users)^a^.

	Early Marijuana Use	Cocaine Use at a Later Age		TotalSubjects
		No	Yes	
Neither Has the Outcome (Pairs = 141)	No	1	0	1
	Yes	1	0	1
Exposed Member Has the Outcome (Pairs = 88)	No	1	0	1
	Yes	0	1	1
Unexposed Member Has the Outcome (Pairs = 21)	No	0	1	1
	Yes	1	0	1
Both Have the Outcome (Pairs = 61)	No	0	1	1
	Yes	0	1	1
Total (Pairs = 311)	No	229	82	311
	Yes	162	149	311

a from Cummings P, McKnight B (2004) Analysis of matched cohort data. Stata J 4: 274–281.

This matched-pair data is in accord with the sparse-data limiting model; there are only two subjects in each stratum but the total number of strata is large. Therefore, I applied the Mantel-Haenszel method in order to pool the strata: 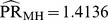 (95% CI: 1.2711∼1.5720). (Both Woolf’s and the standardization method rely on the large-stratum limiting model and are not applicable to this example. The heterogeneity test also relies on the large-stratum limiting model. Therefore, in this example, the assumption of no mechanistic interaction has to be invoked if the strata are to be pooled, but the assumption by itself is not amenable to testing.) This implies a significant ∼1.4 fold decrease in cocaine-naive probability in later years for an early marijuana user when compared with his/her non-marijuana-using co-twin. Ignoring the paired structure of the data, the crude peril ratio for this example is the same as 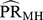 but the variance is larger [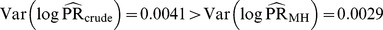].

## Discussion

The estimation of disease-free/survival probabilities and their variances are important first steps for a stratified analysis regarding peril ratios. If censoring (loss to follow up and competing death) occurs in a follow-up study, then the disease-free/survival probabilities can be estimated using the Kaplan-Meier method, and their variances can be estimated using Greenwood’s method [1]. Subsequently, one can proceed to use Woolf’s method in order to obtain the common peril ratio and its variance (under the large-stratum limiting model) as described in this paper. However, a Mantel-Haenszel-typed estimator and its variance for censored data, which is valid under both large-stratum and sparse-data limiting models await further study.

Under the rare-disease assumption that risk of disease is exceedingly low, a log peril ratio can be approximated by the difference between two risks or two odds [13]. For a follow-up study of a rare disease, one therefore has the option of applying existing stratified-analysis techniques in order to estimate the common risk difference [1]. For a case-control study of a rare disease, one should look for a common odds difference, when there is no mechanistic interaction between the exposure under study and the stratifying variable. Further studies are warranted in order to develop stratified-analysis methods regarding odds differences.
